# Primary Extraskeletal Ewing sarcoma of the foot with extensive skeletal and pulmonary metastasis: A rare case report

**DOI:** 10.1016/j.amsu.2022.104752

**Published:** 2022-09-21

**Authors:** Oadi N. Shrateh, Afnan W.M. Jobran, Haneen Owienah, Thaer Sweileh, Mohand Abulihya, Motaz A. Natsheh, Nazeeh Abu-Dayyah

**Affiliations:** a Al-Quds University-School of Medicine, Abu-Dis, East Jerusalem, Palestine; b Radiology Department, Al-Istishari Arab Hospital, Ramallah, West Bank, Palestine; c Pathology Department, Al-Istishari Arab Hospital, Ramallah, West Bank, Palestine; d Orthopedic Surgery Department, Al-Istishari Arab Hospital, Ramallah, West Bank, Palestine

**Keywords:** Extraskeletal Ewing sarcoma, Cutaneous Ewing sarcoma, Foot sarcoma, Case report

## Abstract

**Introduction:**

First -degree cutaneous extraskeletal Ewing's sarcomas (ESs) are incredibly uncommon skin-specific tumors that often present as a single, tiny lesion that is restricted to the mid-to-deep dermis or involves the subcutis. ESs can be clinically and pathologically misdiagnosed because of their rarity and physical resemblance to other cutaneous cancers.

**Case presentation:**

A 47-year-old nonsmoking woman was admitted after being transferred from a nearby hospital to check her right foot pain that had been present for three months and was significantly numbing the same side. Only a few lone cases or brief series are reported in the current literature. The typical description of ESs is that they are tiny masses with positive clinical behavior.

**Discussion:**

Despite being a rather common location, only infrequent and minor ESs of the foot are present. After the recommended operation and subsequent histology analysis, we identified this uncommon sort of tumors.

**Conclusion:**

Although it's rare, it's very important to consider this tumor in the differential diagnosis of foot pain with/without visible and/or palpable cutaneous lesion.

## Highlights

• First -degree cutaneous extraskeletal Ewing's sarcoma is a peculiar soft tissue malignancy occurring in 1 in 5–10 million people.
• Following osteosarcoma, Ewing sarcoma is the second most common primary bone malignancy.
• The patient was treated with surgery followed by chemotherapy.
• This neoplasm should be included in the differential diagnosis of foot pain and/or lesion.

## 1 Introduction

Extraskeletal Ewing sarcoma (EES) is a peculiar soft tissue neoplasm that originally belonged to a group of tiny spherical malignant cells termed ES family of tumors (ESFT). These cells resemble each other in the original neuronal tissue and molecular lineage. Along with EES, ESFT also entails the classic Ewing sarcoma of bone (ESB), which is considered the second most frequent type of primary bone neoplasms in children age group, peripheral primitive neuroectodermal tumor (pPNET) and its variant Askin's tumor of the thoracic wall [1,2]. Although EES was initially identified in 1969 [3]. However, it's yet an enigmatic malignancy in the literature. EES has lower frequency, later-onset and better overall 5-year survival compared to classical ES [4,5]. EES most commonly originates in the proximal thigh, gluteal region and/or upper extremities [6]. Notwithstanding, breast, hepatic and gastrointestinal involvement has been reported [[7], [8], [9]]. EES is dramatically expanding lesion causing compression symptoms such as focal pain [6]. EES diagnosis usually starts with imaging studies. Histology and immunohistochemistry also play an important role in EES detection. Management options of EES may include surgical resection, radiotherapy and/or chemotherapy [1,10]. The vast majority of EES patients are young, with a 20-year median age [11]. Herein, we report a case of EES of the foot with paravertebral involvement in a 47-year-old Palestinian female. This case report has been reported in line with the SCARE Criteria [12].

## 2 Case presentation

**Fig. 1:**
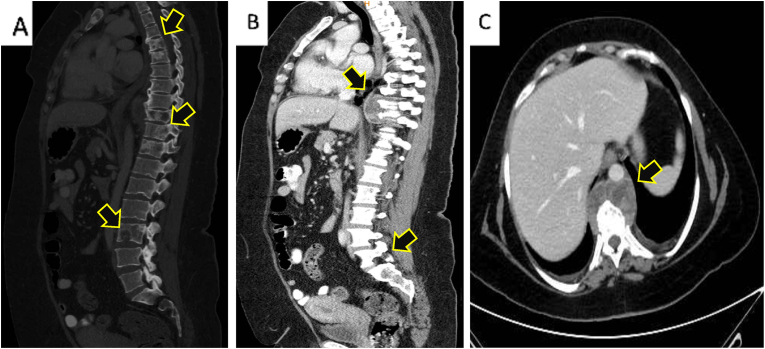
**(A):** is a Computed Tomography (CT) scan with bone window, **(B):** is a CT scan with soft tissue window, and **(C):** is a CT scan with coronal view demonstrating para-spinal soft tissue lesion at the level of T8-T9, (Arrows define the significant findings).

**Fig. 2:**
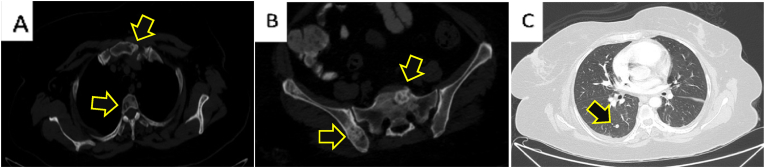
**(A):** is a CT scan showing lytic lesions of the spine and sternum, **(B**): is a CT scan showing sclerotic and lytic lesions of the pelvis, and **(C):** is a CT scan showing metastatic lesion in the right lung, (Arrows define the significant findings).

**Fig. 3:**
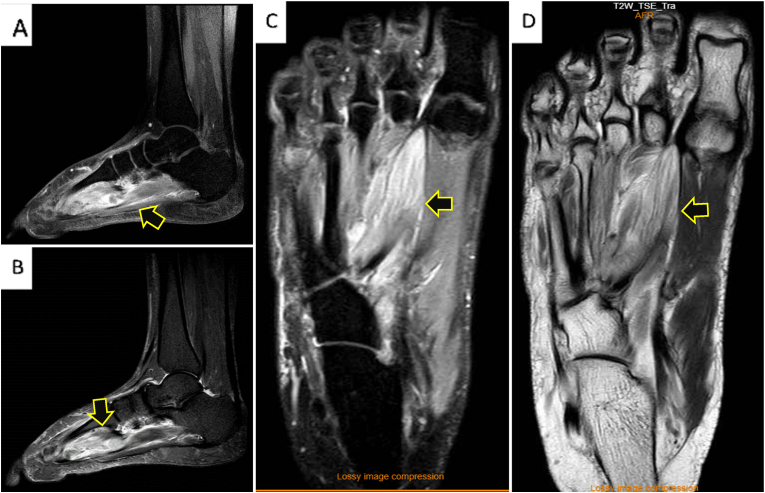
**(A):** is a Magnetic resonance imaging (MRI) of right foot (RF) with T1-weighting**, (B**)**:** is an MRI of RF with Short Tau Inversion Recovery (STIR) sequence, **(C):** is an MRI of RF with Proton density (PD) weighting**,** and **(D):** is an MRI of RF with T2-weighting, (Arrows define the significant findings).

**Fig. 4:**
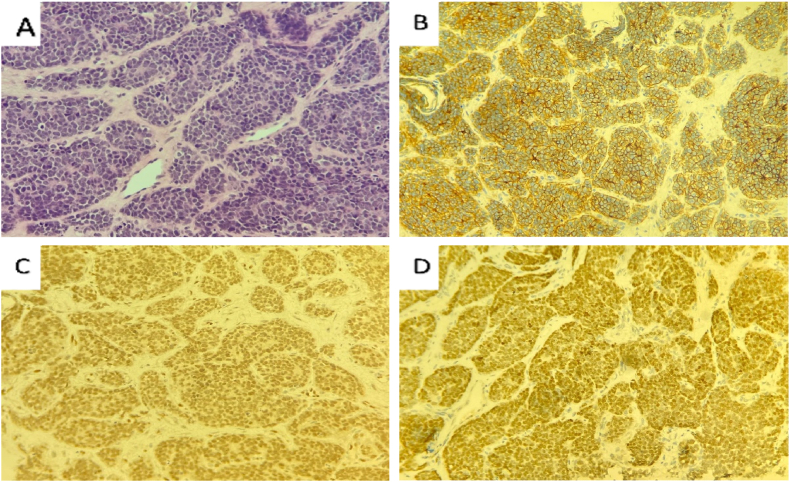
**(A):** H & E stain, **(B):** CD99, **(C):** FLI-1, and **(D):** Anti NKx-2.2.

S.F., A 47-year-old nonsmoker woman transferred to the hospital via ambulance and was afterwards admitted for the assessment of chronic and persistent pain in the right foot for a three-months duration associated with significant numbness on the same side. after that, the patient started to complain of acute-onset sever lower back and pelvic pain. She asserted never suffering pain similar to this before. The patient reported no personal and/or family history of cancer, any acute, repeat, or discontinued medications, any allergies, any genetic or psychosocial issues, and had a free past surgical history. physical examination was unremarkable except for lower back tenderness radiated to both lower limbs and marked lower limb weakness with estimated power tone of 2/5 on the right foot. Neurovascular assessment was insignificant. The patient initially underwent spinal computed tomography (CT) without contrast which revealed multiple enhancing metastatic lesions throughout the spine, the most significant lesions are in the thoracic area, particularly at the level of T8-T10 vertebral bodies and mild pathological compression fractures of T4, T5, T9, T10, T11 and L5 associated with a large anterior paraspinal soft tissue component at the same level (Fig. 1 A, B & C). Whole body computed tomography (CT) with intravenous contrast was performed and showed several mixed lytic and sclerotic lesions in the spinal, sternal, and pelvic bones associated with soft tissue component, the largest one seen at the level of T10. CT scan also detected a nodule measuring about 8 mm in right lower lung lobe (Fig. 2 A, B & C). MRI of right foot demonstrated heterogenous, ill defined, lobulated intra muscular mass measuring approximately 3 x 1.7 × 1.3 cm at the planter aspect of the right foot with irregular peripheral post-contrast enhancement (Fig. 3 A, B, C & D). These findings were suggestive of extraskeletal Ewing Sarcoma and to confirm the diagnosis, Excisional biopsy of right foot lesion was taken and showed marked tumor necrosis with numerous mitotic and apoptotic figures. The tumor cells were arranged in an unusual trabecular pattern with rare pseudorosettes (neuroectodermal differentiation) and immunopositivity for CD99 (diffuse-membranous), FLI-1 (nuclear-strong), anti NKx-2.2, Synaptophysin (weak), CD56 (weak), and CKAE1/AE3 (weak-focal) (Fig. 4 A, B, C & D). The diagnosis of extraskeletal Ewing sarcoma of right foot was established. Intravenous oxycodone was administrated to relive patient's sever pain. The patient initially underwent thoraco-lumber pathological fractures fixation with fusion from T2-L1 level, total surgical resection of the foot mass. The procedure was performed by a consultant at spine surgery and spinal deformities department at a private hospital. The patient was started a chemotherapy regimen consisting of carboplatin and paclitaxel weekly. The patient was followed up for 2 months and She adhered to and tolerated the provided advices; avoiding vigorous exercise and heavy lifting. The patient also had a good tolerance of chemotherapy and pharmacological agents without any reported complications or adverse events.

## 3 Discussion

We discuss an unusual example of extraskeletal Ewing's sarcoma in the foot. Even though it's uncommon, it's crucial to include this tumor in the differential diagnosis of foot discomfort whether or not there is a palpable or visible cutaneous lesion.

Extraskeletal Ewing sarcoma (EES) is a soft tissue sarcoma (STS) that is uncommon, occurring in 1 in 5–10 million people, and makes up only a small percentage of the 12,000 STS that occur annually on average [13]. Few substantial population-based studies that address treatment options and related clinical outcomes, particularly in adults, exist due to the rarity of EES [14]. Without specifically analyzing treatment modalities unique to EES, the largest trials to explore EES compared outcomes to skeletal Ewing sarcoma [13]. In 1975, Angerwall and Enzinger published the first description of them [15]. Since then, Delaplace et alrecent.'s thorough assessment of the literature has found just a few solitary cases or small series [16].

Their typical clinical manifestation is a superficial, single mass that is 2–3 cm in diameter, soft, movable, and occasionally painful, with an average period to evolution of 5 months [17]. A median age upon diagnosis of 17 years [16]. Our reported case of EES of the foot with paravertebral involvement 47-year-old Palestinian female. Numerous auxiliary methods, including aspiration cytology, histochemical stains, immunohistochemistry, electron microscopy, cytogenetics, and molecular genetics of translocations, may be necessary for the diagnosis. Essential criteria for the diagnosis of cutaneous ESs include the histological appearance of small round cells, immunohistochemical positivity for CD99 in a distinctive membrane pattern, and the particular chromosomal translocation involving the gene EWSR1 on chromosome 22q12 [17].

The diagnosis of superficial ES is challenging due to the rarity of these tumors, the relative non-specificity of their histology, and their immunoprofile, and a wide range of differential diagnoses must be taken into account, including Merkel cell carcinoma, cutaneous lymphomas, clear cell sarcoma, malignant primitive neuroectodermal tumor, small cell carcinoma, rhabdomyosarcoma, malignant rhabdoid tumor, and poorly differentiated adnexal Unlike its deep counterpart, they showed a generally positive clinical behavior and had a 91% 10-year survival rate. Due to their tiny size and potential for early discovery and complete surgical removal because of their superficial placement, they most likely prevented metastatic spread in the majority of patients [16]. To check for lung metastases throughout the staging process, the chest CT scan is a crucial step. Bone scintigraphy has historically been used to detect bone metastases. Bone scintigraphy, however, has been substituted in most centers by [18F] fluorodeoxyglucose positron emission tomography (FDG-PET) or a fusion PET-CT since it has proven to be more effective than bone scintigraphy in some studies. The normal work-up also includes a bilateral bone marrow biopsy and aspiration, but many medical professionals are skeptical about their added value in cases when the PET scan is negative [18].

The principles of treatment are currently the same as those for Ewing sarcoma of the bone, including wide surgical resection, being associated with or without chemotherapy and/or radiotherapy, depending on its size and location, even though a conclusive conclusion on the most effective treatment modalities has not yet been clarified because of the extreme rarity of these tumors [16,17]. Chemotherapy has significantly increased patients with localized disease's 5-year overall survival rate from 10% to over 70% [10]. Unfortunately, main metastatic ES, whose 5-year overall survival (OS) is less than 30%, has not seen any improvement. The prognosis for patients who relapse is even worse, with a 5-year event-free survival (EFS) rate of just 10% [19]. Neoadjuvant chemotherapy with multiple agents is often administered for at least 12 weeks before local treatment, which might include surgery, radiation, or a combination of the two. Adjuvant chemotherapy's length and kind are determined by the tumor's response to the chemotherapy, the presence of metastases at the time of diagnosis, and the treatment protocol being used. When possible, it is advised that patients participate in clinical trials [20].

## 4 Conclusion

In conclusion, we describe a peculiar case of foot extraskeletal Ewing's sarcoma. Although its rare, its very important to consider this tumor in the differential diagnosis of foot pain with/without visible and/or palpable cutaneous lesion.

## Provenance and peer review

Not commissioned, externally peer-reviewed.

## Sources of funding for your research

The authors declare that writing and publishing this manuscript was not funded by any organization.

## Ethical approval

This study is exempt from ethical approval in our institution.

## Consent

A written informed consent for the data and picture was taken from the patient and the family and available upon request from the Editor-in-Chief.

## Authors contribution

Writing the manuscript: Oadi N. Shrateh, Afnan W.M. Jobran Imaging description: Haneen Owienah, Oadi N. Shrateh, Thaer Sweileh, Mohand Abulihya, Motaz A. Natsheh Reviewing & editing the manuscript: Nazeeh Abu-Dayyah, Oadi N. Shrateh, Afnan W.M. Jobran.

## Registration of research studies

Not Applicable.

## Guarantor

Oadi N. Shrateh.

## Declaration of competing interest

The authors declare that there is no conflict of interest regarding the publication of this article.
